# Comparison of aerosol delivery across combinations of drug delivery interfaces with and without concurrent high-flow nasal therapy

**DOI:** 10.1186/s40635-019-0245-2

**Published:** 2019-04-03

**Authors:** Gavin Bennett, Mary Joyce, Elena Fernández Fernández, Ronan MacLoughlin

**Affiliations:** Aerogen Limited, IDA Business Park, Dangan, Galway, Ireland

**Keywords:** Aerosol, Vibrating mesh nebulizer, High-flow nasal therapy, Nasal cannula, Facemask, Mouthpiece, Aerosol chamber

## Abstract

**Background:**

Current clinical practice during high-flow nasal therapy (HFNT) involves utilization of a nasal cannula to provide humidification, with a facemask placed over the cannula to deliver aerosol. Few studies have compared aerosol delivery across various delivery interfaces during HFNT. The objective of this study was to address this gap in the literature and evaluate aerosol delivery using two nebulizer types across different drug delivery interfaces, nasal cannula, facemask, and mouthpiece, during simulated adult HFNT.

**Methods:**

A facemask or mouthpiece and/or a nasal cannula were positioned on an anatomically correct adult head model. The head model was connected to a breathing simulator via a collection filter. Both healthy breathing pattern and distressed breathing patterns were utilized. Aerosol dose was determined by quantifying the mass of drug captured on a filter positioned distal to the trachea.

**Results:**

During simulated healthy breathing, a significantly greater aerosol dose was observed when the vibrating mesh nebulizer (VMN) was integrated with HFNT alone, supplying aerosol and humidified air simultaneously (2.88 ± 0.15%), as opposed to using with a facemask (0.33 ± 0.07%, 1.62 ± 0.46%, and 1.07 ± 0.25% at 0 L/min (LPM), 2LPM, and 6LPM, respectively) or mouthpiece (0.56 ± 0.13%, 2.16 ± 0.06%, and 1.82 ± 0.41% at 0LPM, 2LPM, and 6LPM). In addition, aerosol delivery was also significantly greater when the VMN was integrated into simulated HFNT (2.88 ± 0.15%), in comparison with using the jet nebulizer (JN) with a facemask (0.82 ± 0.16%) or a mouthpiece (0.86 ± 0.11%). During simulated distressed breathing, a significantly greater aerosol dose was observed when the VMN was integrated with HFNT, supplying aerosol and humidified air simultaneously (6.81 ± 0.45%), compared with using a facemask (0.86 ± 0.04%, 2.96 ± 0.26%, and 4.23 ± 0.93% at 0LPM, 2LPM, and 6LPM) or mouthpiece (0.73 ± 0.37%, 0.97 ± 0.20%, and 3.11 ± 0.53% at 0LPM, 2LPM, and 6LPM, respectively). Aerosol delivery was also greater when the VMN was integrated into HFNT (6.81 ± 0.45%), in comparison with using the JN with a facemask (5.72 ± 0.71%) or a mouthpiece (0.69 ± 0.53%). Furthermore, across all drug delivery interfaces, and in line with previous reports, aerosol delivery was greater during simulated distressed breathing, in comparison with simulated healthy adult breathing.

**Conclusions:**

This article will be of considerable benefit in enhancing the understanding of aerosol delivery during HFNT, an increasingly adopted therapeutic intervention by healthcare professionals.

## Background

High-flow nasal therapy (HFNT) is a means of delivering heated humidified air to patient airways that facilitates higher gas flows than conventional low-flow therapy [1]. HFNT provides flow rates that equal or exceed inspiratory flow and reduces the inspiratory resistance associated with the nasopharynx, thus reducing the work of breathing [2, 3]. The mounting clinical evidence in combination with its ease of use and patient tolerability has resulted in increasing adoption of HFNT, with a particular interest in concurrent aerosol delivery during HFNT [4–8]. Examples of medications that have been delivered concurrently via aerosol include bronchodilators and mucolytics in the treatment of chronic and acute episodes of respiratory illness, such as chronic obstructive pulmonary disease (COPD) and asthma [9].

Several factors have been reported to affect the quantity of aerosol exiting the cannula during HFNT. These include the rate of gas delivered, size of the nasal prongs, humidification system, size of the aerosol droplets, and the type and position of aerosol generator [10–12]. Previously, our group reported a systematic approach to determine the conditions required to yield an optimal emitted dose, thus becoming available for inhalation during HFNT. The findings in that study clearly indicate that in order to optimize the amount of aerosol exiting the nasal prongs during HFNT, it is necessary for the gas flow rate to be low and the input droplet size to be small, while the nebulizer should be positioned immediately after the humidification chamber [13]. In a recent scintigraphy study, Dugernier et al. demonstrated in vivo that lung deposition was significantly greater while using a vibrating mesh nebulizer (VMN), in comparison with a jet nebulizer (JN) during adult HFNT [14].

There are various types of drug delivery modalities utilized during HFNT, including facemasks, mouthpieces, and nasal cannula. Morgan et al. showed that infants with acute bronchiolitis tolerated aerosolized β-agonist therapy better during HFNT than with a facemask [15]. Ari et al. showed that aerosol delivery with a mouthpiece was more efficient than a standard aerosol mask during simulated adult and pediatric breathing, using jet and mesh nebulizers [16]. Our group previously demonstrated mouthpiece-mediated aerosol delivery via an aerosol chamber during concurrent HFNT in vitro, using a vibrating mesh nebulizer. The largest aerosol dose was observed with a 6LPM aerosol chamber gas flow and a 10LPM HFNT system [17].

Current clinical practice for aerosol therapy during HFNT involves utilization of a nasal cannula to provide humidification, with a facemask placed over the cannula to deliver aerosol. Medications are generally administered with a JN connected to a facemask, despite the poor tolerability of this interface and low lung deposition [18, 19]. The use of a JN in patients receiving HFNT may require the discontinuation of respiratory support to release the nasal route and consequently increased discomfort for patients, hence the reported use of a JN with facemask over the nasal cannula [11, 20, 21]. The inclusion of a JN in a HFNT circuit may also be contraindicated on the basis that it may interfere with oxygen levels and gas flows. To date, there has been no report that has compared aerosol delivery across these various modalities during HFNT. Therefore, the main objective of this study was to address this gap in the current literature and evaluate aerosol delivery across combinations of different drug delivery interfaces such as nasal cannula, facemask, and mouthpiece during simulated adult HFNT, using two prevalent nebulizer types.

## Methods

### High-flow nasal therapy circuit

The Optiflow™ system (AIRVO 2, Fisher and Paykel Healthcare, Auckland, New Zealand) was employed to supply humidified air. The AIRVO 2 system features a humidifier with an integrated flow source and was used in conjunction with the provided nebulizer adapter. An adult breathing circuit (P/N: 900PT552) was used with an adult nasal cannula (P/N: OPT + 944). In line with the recommendations of the FLORALI trial, all testing was completed with a HFNT gas flow rate of 50 L/min [22].

### Nebulizers

Experiments were performed using a vibrating mesh nebulizer (VMN) (Aerogen Solo, Aerogen Ltd., Galway, Ireland) and a jet nebulizer (JN) (Cirrus 2, Intersurgical, Wokingham, UK). The JN was operated with the standard driving gas flow rate of 8LPM. The nebulizer performance characteristics of the JN are outlined in terms of average particle size (3.3 μm). The nebulizer performance characteristics of the VMN, measured using laser diffraction (Spraytec, Malvern Instruments, Malvern, UK) as previously described [23] are outlined in terms of average droplet size (4.57 ± 0.07 μm Volumetric Median Diameter, VMD) and aerosol output rate (0.35 ± 0.00 mL/min).

### Interfaces

**Fig. 1:**
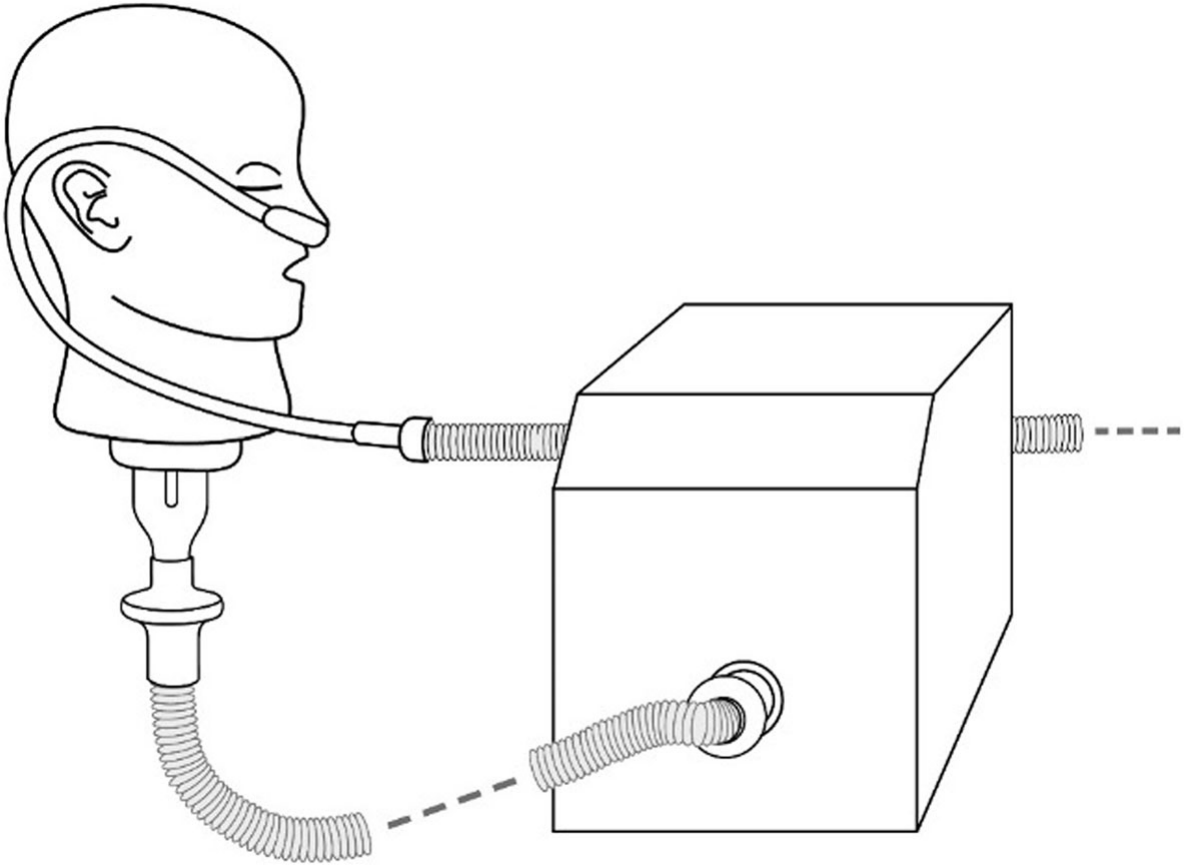
High-flow nasal cannula was positioned on an adult head model, which was connected to a breathing simulator via a collecting filter. The VMN was positioned at the humidification chamber

**Fig. 2:**
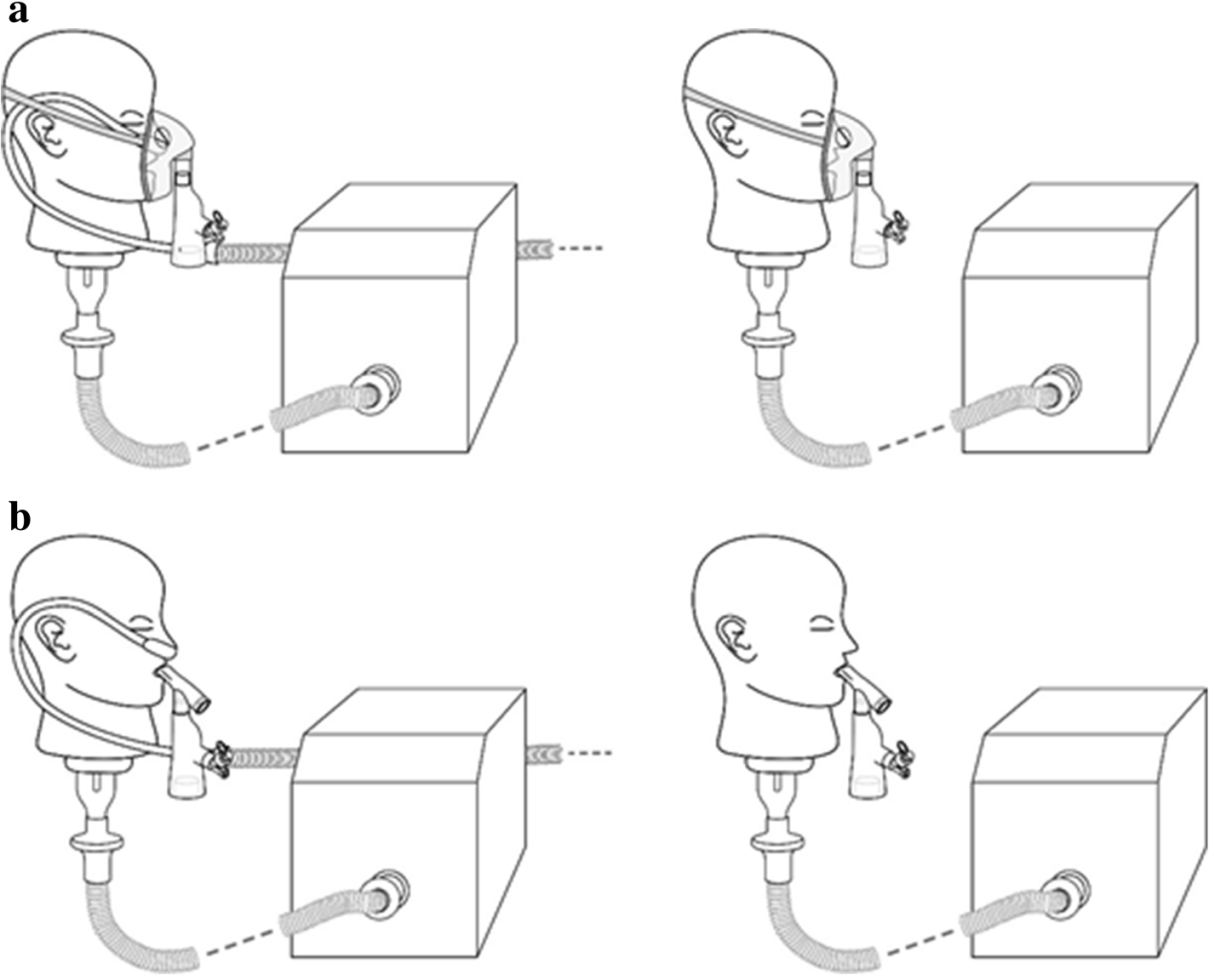
**a** A facemask, utilized with an aerosol chamber positioned on an adult head model, was connected to a breathing simulator via a collecting filter, with and without concurrent HFNT. **b** A mouthpiece, utilized with an aerosol chamber positioned on an adult head model, was connected to a breathing simulator via a collecting filter, with and without concurrent HFNT. The VMN was positioned in the aerosol chamber

**Fig. 3:**
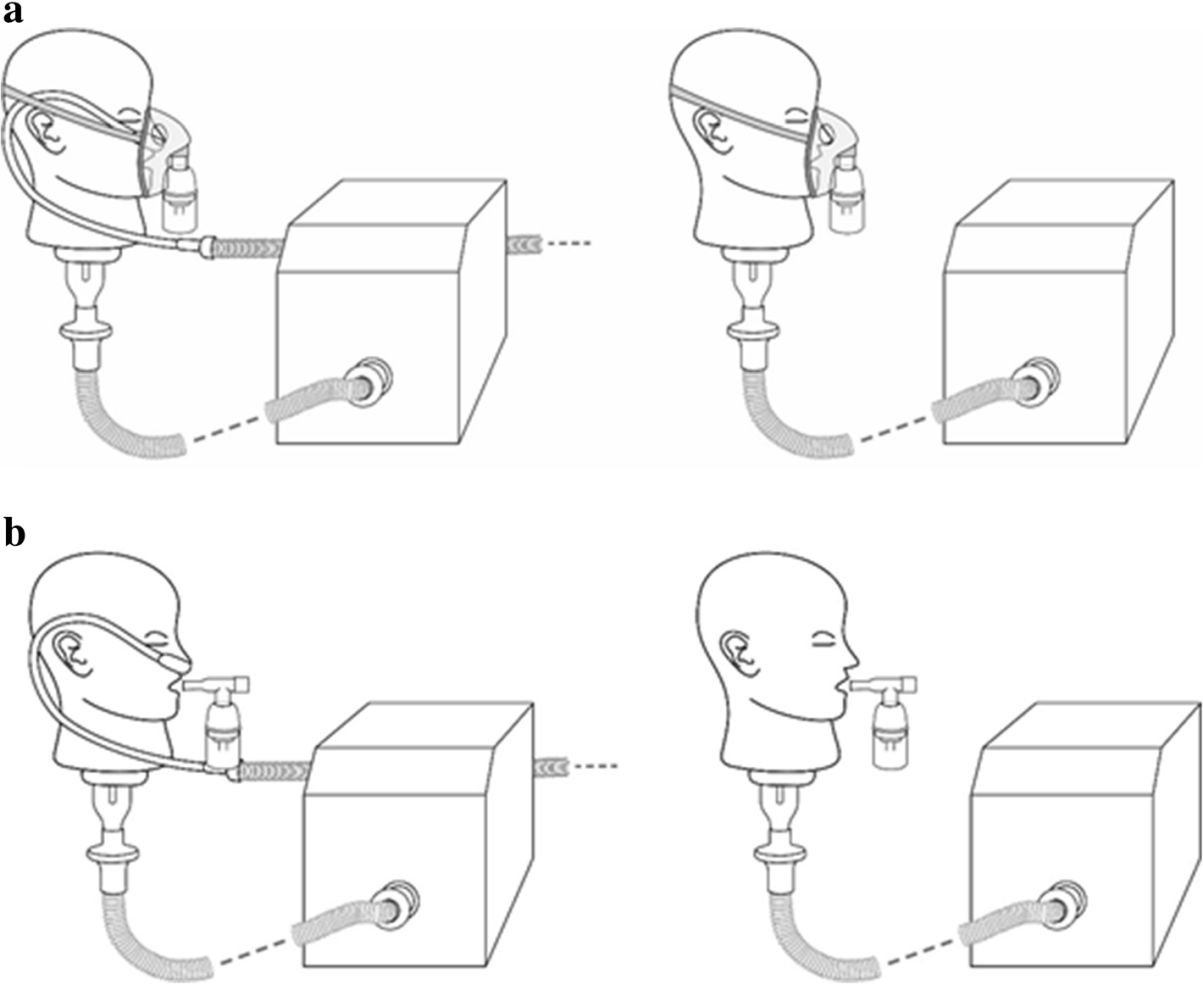
**a** A facemask, positioned on an adult head model, was used with a jet nebulizer and was connected to a breathing simulator via a collecting filter, with and without concurrent HFNT. **b** A mouthpiece was used with a JN and was connected to a breathing simulator via a collecting filter, with and without concurrent HFNT

For HFNT with a nasal cannula, the VMN was positioned at the humidification chamber (Fig. 1). A facemask (Salter Labs, Chicago, USA) was utilized with an aerosol chamber (Aerogen Ultra, Aerogen Ltd., Galway, Ireland) in combination with the VMN, with and without concurrent HFNT (Fig. 2a). A mouthpiece was utilized with the Aerogen Ultra aerosol chamber in combination with the VMN, with and without concurrent HFNT (Fig. 2b). A facemask (Intersurgical Ecolite mask, Intersurgical, Wokingham, UK) was used in combination with a JN (Cirrus 2, Intersurgical, Wokingham, UK), with and without concurrent HFNT (Fig. 3a). A mouthpiece (Intersurgical, UK) was used in combination with a JN (Cirrus 2, Intersurgical, UK), with and without concurrent HFNT (Fig. 3b).

### Aerosol dose

A facemask or mouthpiece and/or a nasal cannula were positioned on a previously described 3D-printed anatomically correct adult head model [17]. The head model was connected to a breathing simulator (Ingmar ASL 5000, Ingmar Medical, Pittsburgh, USA) via a collecting filter (RespirGard II 303, Baxter, Ireland). A healthy adult breathing pattern (tidal volume 500 mL, breath rate 15 BPM, and inspiratory to expiratory ratio 1:1) [20, 23] and a distressed adult breathing pattern (tidal volume 750 mL, breath rate 30 BPM, and inspiratory to expiratory ratio 1:1) [20] were used. Aerosol dose was determined by quantifying the mass of drug captured on a filter positioned distal to the trachea. The humidifier was powered on and allowed to come to temperature (37 °C), and a 2-mL dose of albuterol sulfate (2 mg/mL) (GlaxoSmithKline Ltd., Dublin, Ireland) was nebulized. Albuterol was used as it is a commonly nebulized formulation used in the characterization of aerosol drug delivery systems and is specified for use as a tracer aerosol in the international standard ISO 27427:2013 [24]. Further, it is a commonly used bronchodilator in bronchospasm caused by asthma and chronic obstructive pulmonary disease and so was considered an appropriate choice of aerosol tracer. At the end of each dose administration, the drug captured on a filter was eluted using 10 mL of deionized water. The mass of drug was quantified by means of UV spectrophotometry at a wavelength of 276 nm and interpolation on a standard curve of albuterol sulfate concentrations (from 200 μg/mL to 3.125 μg/mL). Results for aerosol dose were expressed as the percentage of the nominal dose initially placed in the nebulizer’s medication cup.

### Statistical data analysis

Results are expressed as mean ± standard deviation aerosol dose (percentage). Student’s *t* tests were conducted to establish if the aerosol dose varied significantly across different drug delivery interfaces, with and without concurrent HFNT, while using two nebulizer types. *p* values of < 0.05 were considered statistically significant. The experiments were repeated three times independently (*n* = 3) for each test scenario.

## Results

### Healthy adult breathing

**Fig. 4:**
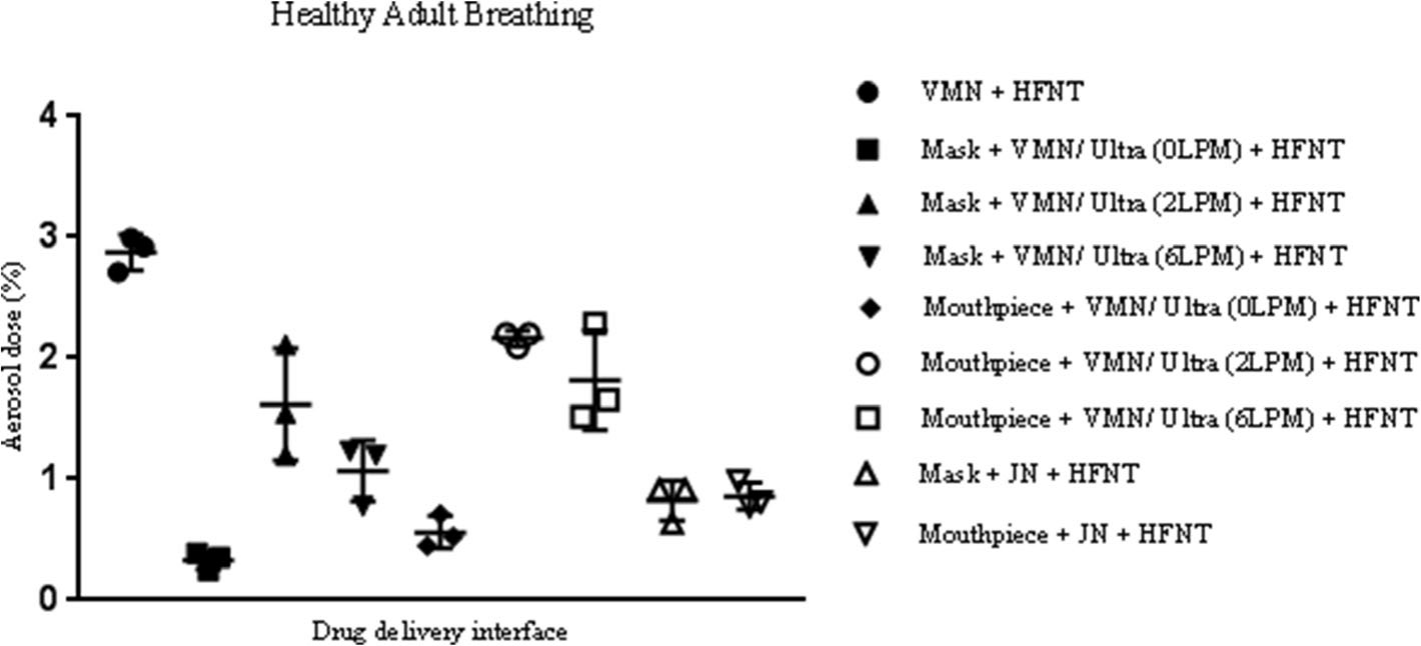
Illustration of aerosol dose (percentage) for each delivery modality with concurrent HFNT, during simulated healthy adult breathing. The values represented are mean ± standard deviation (expressed in percentage) of three independent experiments

The mean ± standard deviation values of aerosol dose (percentage) during simulated healthy adult breathing are outlined in Table 1 and Fig. 4. Aerosol delivery was significantly greater without concurrent HFNT when using both the VMN and JN in combination with a facemask or mouthpiece. When using the VMN with a nasal cannula, aerosol dose was significantly greater when aerosol and humidified air were supplied simultaneously through a nasal cannula using the AIRVO 2 (2.88 ± 0.15%), compared with a facemask (0.33 ± 0.07%, 1.62 ± 0.46%, and 1.07 ± 0.25% with supplemental gas flow rates of 0LPM (no supplemental oxygen), 2LPM, and 6LPM, *p* value < 0.0001, 0.0109, and 0.0004, respectively) or mouthpiece (0.56 ± 0.13%, 2.16 ± 0.06%, and 1.82 ± 0.41% with supplemental gas flow rates of 0LPM (no supplemental oxygen), 2LPM, and 6LPM, *p* value < 0.0001, 0.0015, and 0.0140, respectively). In addition, aerosol delivery was significantly greater when the VMN was integrated into simulated HFNT (2.88 ± 0.15%), in comparison with using the JN with a facemask (0.82 ± 0.16%, *p* value < 0.0001) or a mouthpiece (0.86 ± 0.11%, *p* value < 0.0001). For the most part, increasing supplemental gas flow rates through the aerosol chamber in combination with a facemask and mouthpiece were associated with an increased aerosol dose.

**Table 1:** Results of aerosol dose across different drug delivery interfaces during simulated healthy adult and distressed breathing. *LPM* liters per minute, *N/A* not applicable. VMN does not require supplemental gas flow for normal operation. The values represented are mean ± standard deviation (expressed in percentage) of three independent experiments

	Supplemental gas flow rate (LPM)	Aerosol dose (%) Healthy adult breathing	Aerosol dose (%) Distressed adult breathing
VMN + HFNT at 50LPM	N/A	2.88 ± 0.15	6.81 ± 0.45
Mask + VMN/Ultra	0LPM	3.43 ± 0.62	28.76 ± 1.72
	2LPM	29.93 ± 0.46	35.47 ± 1.81
	6LPM	22.44 ± 0.63	36.21 ± 0.78
Mask + VMN/Ultra + HFNT at 50LPM	0LPM	0.33 ± 0.07	0.86 ± 0.04
	2LPM	1.62 ± 0.46	2.96 ± 0.26
	6LPM	1.07 ± 0.25	4.23 ± 0.93
Mouthpiece + VMN/Ultra	0LPM	0.63 ± 0.07	1.92 ± 1.12
	2LPM	28.72 ± 1.24	21.37 ± 0.78
	6LPM	31.52 ± 0.35	28.46 ± 0.38
Mouthpiece + VMN/Ultra + HFNT at 50LPM	0LPM	0.56 ± 0.13	0.73 ± 0.37
	2LPM	2.16 ± 0.06	0.97 ± 0.20
	6LPM	1.82 ± 0.41	3.11 ± 0.53
Mask + JN	8LPM	6.13 ± 0.09	9.07 ± 0.26
Mask + JN + HFNT at 50LPM	8LPM	0.82 ± 0.16	5.72 ± 0.71
Mouthpiece + JN	8LPM	12.68 ± 1.16	12.90 ± 2.52
Mouthpiece + JN + HFNT at 50LPM	8LPM	0.86 ± 0.11	0.69 ± 0.53

### Distressed adult breathing

**Fig. 5:**
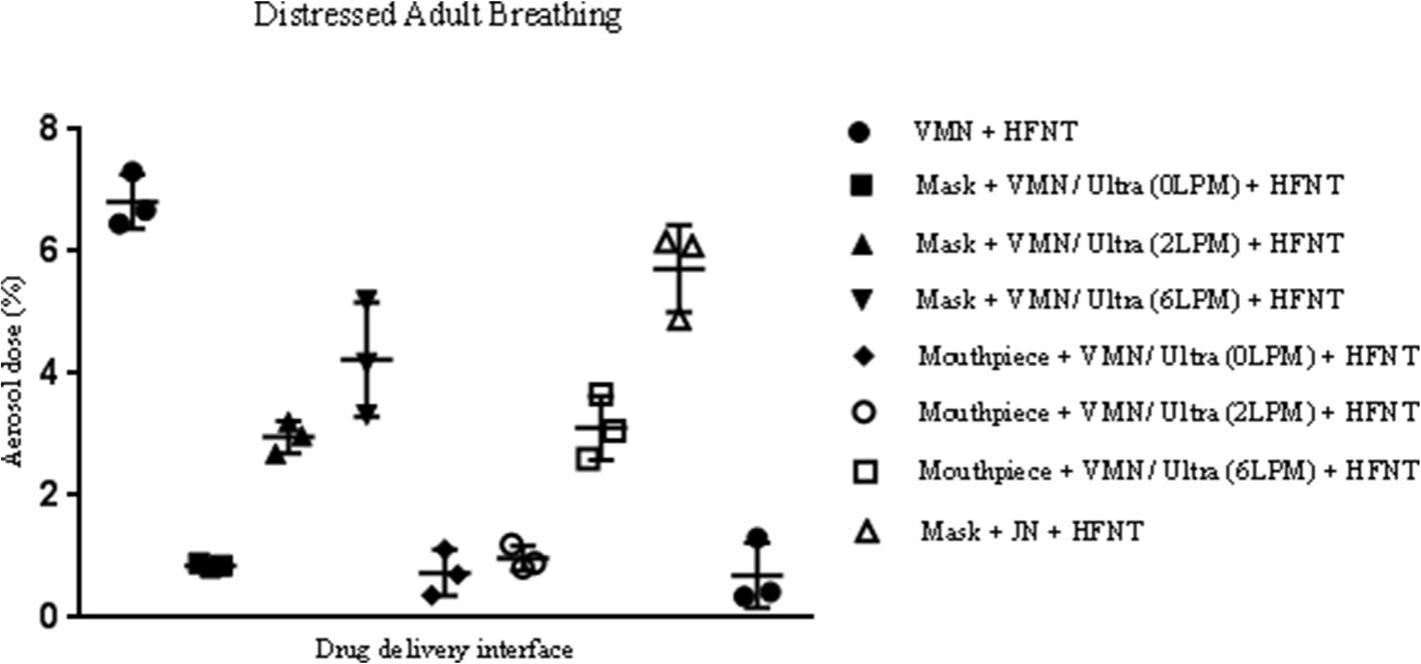
Illustration of aerosol dose (percentage) for each delivery modality with concurrent HFNT, during simulated distressed adult breathing. The values represented are mean ± standard deviation (expressed in percentage) of three independent experiments

The mean ± standard deviation values of aerosol dose (percentage) during simulated adult breathing are outlined in Table 1 and Fig. 5. Aerosol delivery was consistently greater across all interfaces during simulated distressed breathing, compared with healthy breathing. Aerosol delivery was significantly greater without concurrent HFNT, when using the VMN and JN in combination with a facemask or mouthpiece. While utilizing the VMN with HFNT, aerosol dose was significantly greater when aerosol and humidified air were supplied simultaneously through a nasal cannula using the AIRVO 2 (6.81 ± 0.45%), compared with facemask (0.86 ± 0.04%, 2.96 ± 0.26%, and 4.23 ± 0.93% with supplemental gas flow rates of 0LPM (no supplemental oxygen), 2LPM, and 6LPM, *p* value < 0.0001, 0.0002, and 0.0123, respectively) or mouthpiece (0.73 ± 0.37%, 0.97 ± 0.20%, and 3.11 ± 0.53% with supplemental gas flow rates of 0LPM (no supplemental oxygen), 2LPM, and 6LPM, *p* value < 0.0001, < 0.0001, and 0.0008, respectively). Aerosol delivery was greater when the VMN was integrated into HFNT (6.81 ± 0.45%), in comparison with using the JN with a facemask (5.72 ± 0.71%, *p* value 0.0860, not statistically significant) or a mouthpiece (0.69 ± 0.53%, *p* value 0.0001). Increasing supplemental gas flow rates through the aerosol chamber in combination with a facemask or mouthpiece were associated with an increased aerosol dose.

## Discussion

This is the first study to evaluate aerosol delivery across various drug delivery interfaces during simulated HFNT. A significantly greater aerosol dose was observed when the VMN was integrated with HFNT, supplying aerosol and humidified air simultaneously, as opposed to using a facemask or mouthpiece with the VMN or JN. Furthermore, across drug delivery interfaces, aerosol delivery was greater during simulated distressed breathing, in comparison with simulated healthy adult breathing. Efficient aerosol delivery to the lungs during HFNT is challenging due to the high-velocity gas flows utilized, which may promote aerosol deposition in the nasal passages [20]. Consequently, aerosol dose (percentage) results were relatively low.

Aerosol delivery was significantly greater without concurrent HFNT, when using the VMN and JN in combination with either a facemask or mouthpiece, during simulated healthy and distressed breathing. However, in situations where HFNT is not discontinued, a reduction in aerosol delivery may be expected on the basis of higher gas flow rates, humidification, and potential interference of the nasal cannula with aerosol transit. Humidification during HFNT is essential for proper function of the epithelial lining and is an accepted standard of care. Without humidification, unidirectional inspiratory nasal airflow may lead to the drying of mucosa and release of inflammatory mediators [25]. Therefore, two types of humidification device, heated humidifier and heat and moisture exchanger, are utilized during short-term and long-term non-invasive ventilation [26].

When using the VMN with a nasal cannula, aerosol dose was significantly greater when aerosol and humidified air were supplied simultaneously through a nasal cannula, compared with a facemask or mouthpiece in line in the VMN or JN. This finding was consistent across simulated healthy and distressed adult breathing. The AIRVO 2 system features a humidifier with an integrated flow source and was used in conjunction with a previously mentioned nebulizer adapter [27]. The elimination of added interfaces (facemask or mouthpiece) in this integrated therapy likely explains a reduction in aerosol losses. Furthermore, differences in the point of entry of aerosol into humidified/ non-humidified air across the various delivery modalities may also be a contributing factor, where aerosol is entrained in the gas flow in the most efficient manner, thereby avoiding impactional losses within the circuit and patient interface. Clinical studies demonstrate that the connection of a nebulizer to a HFNT circuit enables continuous nebulization, therefore improving efficiency and tolerance of the therapy [20, 28, 29]. Valencia and colleagues recently reported that the use of a nebulizer incorporated into HFNT results in an increased level of comfort and satisfaction compared to the use of a conventional JN in bronchiolitis patients who require HFNT [21]. Similarly, Morgan et al. showed infants with acute bronchiolitis tolerated aerosolized β-agonist therapy better during HFNT than with a facemask [15].

Aerosol delivery efficiency was greater during simulated distressed breathing, in comparison with healthy adult breathing. This is the first study to assess the effect of breathing pattern on aerosol delivery across the various potential nebulizer/HFNT combinations. Increasing tidal volume was associated with a greater aerosol dose. This is consistent with the findings of Bhashyham et al. who showed that aerosol output dose increased from 18.6% with a tidal volume of 150 mL to 25.4% with a tidal volume of 300 mL during simulated HFNT [10]. An increased breath rate was associated with a greater aerosol dose. This is similar to results reported by Reminiac et al. where the respirable mass of drug was significantly higher during simulated respiratory distress [20]. Dailey et al. showed that with a distressed breathing pattern, aerosol delivery was greater at 30 and 50 L/min than with a quiet breathing pattern [30].

For the most part, increasing supplemental gas flow rates through the aerosol chamber in combination with a facemask or mouthpiece were associated with an increased aerosol dose. Our group previously showed this with a mouthpiece during simulated adult HFNT [17].

### Study limitations

The intention of this study was to assess how inhaled aerosol efficiency is affected by various drug delivery interfaces during simulated breathing, using a 3D-printed anatomically correct airway model. Future studies are required to investigate how drug delivery interfaces affect tracheal deposition in vivo through scintigraphy technique.

HFNT is often used to deliver bronchodilators and administer oxygen for the treatment of acute exacerbations of COPD and acute asthma [20]. In such patients, the I:E ratio may be different from the one utilized in this study (1:1), with the expiratory phase likely being more prolonged. Aerosol delivery efficiency will undoubtedly be affected by such changes in the I:E ratio. The intention of this study was not to replicate specific disease states, but rather to evaluate aerosol delivery across various drug delivery interfaces with breathing patterns that have been previously employed in in vitro studies to assess aerosol delivery during simulated HFNT.

Furthermore, it should be noted that UV spectrophotometry measurements were not blinded.

## Conclusion

This study established the effects of various drug delivery interfaces on the quantity of aerosol that could potentially reach the lung during simulated HFNT. During simulated healthy adult breathing, a significantly greater aerosol dose was observed when the VMN was integrated with HFNT, supplying aerosol and humidified air simultaneously, as opposed to using a facemask or mouthpiece with the VMN or JN. During distressed adult breathing, a significantly greater dose was observed when the VMN was integrated with HFNT, compared with using a facemask or mouthpiece. Aerosol delivery was also greater when the VMN was integrated into HFNT, in comparison with using the JN with a facemask (not statistically significant) or a mouthpiece. This article will be of considerable benefit in improving the understanding of aerosol delivery during HFNT, an increasingly adopted therapeutic intervention by clinicians and healthcare professionals nowadays.

## Abbreviations

HFNT: High-flow nasal therapy
VMN: Vibrating mesh nebulizer
JN: Jet nebulizer
